# Optical Surface Management System and BladderScan for Patient Setup During Radiotherapy of Postoperative Prostate Cancer

**DOI:** 10.1155/2024/3573796

**Published:** 2024-09-04

**Authors:** Hao Chen, Yandong Liu, Songbin Qin, Guanghui Gan

**Affiliations:** Department of Radiation Oncology First Affiliated Hospital of Soochow University, Suzhou 215000, China

**Keywords:** BladderScan, CBCT, Dice similarity coefficient, optical surface imaging, real-time setup

## Abstract

**Background:** The precision of postoperative prostate cancer radiotherapy is significantly influenced by setup errors and alterations in bladder morphology. Utilizing daily cone beam computed tomography (CBCT) imaging allows for the correction of setup errors. However, this naturally leads to the question of the issue of peripheral dose and workload. Thus, a zero-dose, noninvasive technique to reproduce the bladder volume and improve patient setup accuracy was needed.

**Purpose:** The aim of this study is to investigate if the setup method by combining Optical Surface Management System (OSMS) and BladderScan can improve the accuracy of setup and accurately reproduce the bladder volume during radiotherapy of postoperative prostate cancer and to guide CTV-PTV margins for clinic.

**Method:** The experimental group consisted of 15 postoperative prostate cancer patients who utilized a setup method that combined OSMS and BladderScan. This group recorded 103 setup errors, verified by CBCT. The control group comprised 25 patients, among whom 114 setup errors were recorded using the conventional setup method involving skin markers; additionally, patients in this group also exhibited spontaneous urinary suppression. The errors including lateral (Lat), longitudinal (Lng), vertical directions (Vrt), Pitch, Yaw, and Roll were analyzed between the two methods. The Dice similarity coefficient (DSC) and volume differences of the bladder between CBCT and planning CT were compared as the bladder concordance indicators.

**Results:** The errors in the experimental group at Vrt, Lat, and Lng were 0.17 ± 0.12, 0.22 ± 0.17, and 0.18 ± 0.12 cm, and the control group were 0.25 ± 0.15, 0.31 ± 0.21, 0.34 ± 0.22 cm. The rotation errors of Pitch, Roll, and Yaw in the experimental group were 0.18 ± 0.12°, 0.11 ± 0.1°, and 0.18 ± 0.13°, and in the control group, they were 0.96 ± 0.89°, 1.01 ± 0.86°, and 1.02 ± 0.84°. The DSC and volume differences were 92.52 ± 1.65% and 39.99 ± 28.75 cm^3^ in the patients with BladderScan, and in the control group, they were 62.98 ± 22.33%, 273.89 ± 190.62 cm^3^. The *P* < 0.01 of the above performance indicators indicates that the difference is statistically significant.

**Conclusion:** The accuracy of the setup method by combining OSMS and BladderScan was validated by CBCT in our study. The method in our study can improve the setup accuracy during radiotherapy of postoperative prostate cancer compared to the conventional setup method.

## 1. Introduction

To mitigate patient setup errors, it is a standard practice to extend the planning target volume (PTV) from the clinical target volume (CTV), thereby preventing inadvertent underexposure of the target volume [1, 2]. Indeed, cone beam computed tomography (CBCT) is regarded as the gold standard for identifying patient setup errors and internal organ variations. The margins for clinical target volume to planning target volume (CTV-PTV) adjustments are typically established through retrospective analysis of error data obtained from CBCT scans during radiotherapy [3]. Currently, radiotherapy stands as a primary approach for postoperative prostate cancer treatment. Predominantly, patient immobilization strategies involve utilizing vacuum bags and establishing the isocenter using skin markers. During subsequent fractionated treatments, patient positioning was achieved through a two-dimensional alignment of laser beams with skin markers. However, this method is prone to setup errors; particularly, it is difficult to correct the errors caused by patient rotation and weight change. Typically, the CTV-PTV margins for postoperative prostate cancer range between 0.5 and 0.8 cm. But a larger PTV will inevitably bring serious side effects to the surrounding normal tissues. Consequently, the straightforward approach of CTV-PTV expansion appears inadequate to align with the requirements of contemporary precision radiotherapy.

Lately, significant research attention has been directed towards surface-guided radiotherapy (SGRT), encompassing optical surface imaging (OSI) systems, clinical applications of SGRT, and OSI-based clinical research [4]. OSI technology provides real-time 3D surface imaging with a broad field of view (FOV). It is well suited for in-room interactive patient setup and real-time motion monitoring at any couch rotation during radiotherapy [5, 6]. The use of OSI allows for the mitigation of patient setup errors and the management of patient movement. This approach has been validated, demonstrating an agreement of 0.1 cm when compared to the conventional patient setup involving skin markers [7]. The Optical Surface Management System (OSMS) for patient setup of pelvic targets radiotherapy has attractive features verified by CBCT [8]. Nonetheless, OSMS does not address the challenge of inconsistent bladder filling observed during fractionated treatment for postoperative prostate cancer. This issue can result in an uneven distribution of radiation dosage, subsequently influencing genitourinary toxicity outcomes.

Intensity-modulated radiation therapy (IMRT) and volumetric modulated arc therapy (VMAT) are widely recognized radiotherapy techniques frequently employed in postoperative prostate cancer treatment. The inherent inverse planning of IMRT and VMAT results in a highly conformal delivery of the prescribed dose to the target area, resembling a “surgical” approach to radiotherapy [9, 10]. However, a pressing concern, stemming from the heightened conformal nature of these techniques, is the reliance on an assumption of rigid body conditions, where the target and bladder remain stationary—an ideal scenario that requires urgent attention [11]. This circumstance can lead to inadequate dosage reaching the target and excessive dosage affecting the bladder due to inconsistencies in bladder filling as compared to the planning CT, even when a robust immobilization technique is employed to ensure the reproducibility of patient positioning. The traditional approach involved instructing patients to consume a consistent amount of water and maintain a uniform wait time before each treatment session [12]. However, clinical observations indicate significant variations in bladder volume owing to individual differences. CBCT is frequently employed in radiotherapy to rectify errors arising from patient setup. Through CBCT images fused with CT scans, physicians can assess bladder filling status. If inadequate, patients are requested to increase water intake. Nevertheless, this approach is time intensive and exposes patients to additional ionizing radiation through repeated CBCT scans. The ultrasonic BladderScan device presents appealing attributes for attaining a consistent full bladder state. It aids patients in achieving adequate bladder filling, resulting in larger bladder volumes and a reduced failure rate—a validation corroborated by CBCT imaging [13]. The utilization of the BladderScan device has garnered substantial attention in existing literature [14]. However, studies that integrate OSMS and BladderScan for postoperative prostate cancer radiotherapy are scarce.

The aim of this study is to investigate the reliability and accuracy of OSMS combined with BladderScan in guiding radiotherapy for postoperative prostate cancer. The efficacy of this method was evaluated through a comparison with the conventional patient positioning technique involving skin markers. The findings from this study will provide valuable insights to the radiation therapy department regarding the suitability of implementing this approach for pretreatment guidance in postoperative prostate cancer cases.

## 2. Materials and Methods

### 2.1. Patients

A retrospective analysis was conducted on 40 patients with postoperative prostate cancer who received radiotherapy at the Department of Radiotherapy, First Affiliated Hospital of Soochow University, between December 2021 and March 2022. The postoperative prostate patients were consecutively recruited, and the setup method was randomly assigned. None of the patients had metastatic disease, and all patients could follow instructions to fill the bladder. The selected patients ranged in age from 55 to 68 years (median age: 60), weight from 63 to 82 kg (median weight: 73), and height from 162 to 175 cm (median height: 167), and all patients received a treatment prescription of 66 Gy in 33 fractions. Among them, 15 patients were assigned to experimental group A, where BladderScan was used to ensure bladder volume consistency and OSMS was used to patient setup. The remaining 25 patients were assigned to control group B, where patients prepared their bladder volume spontaneously by urine withholding, and the patient setup utilized the skin markers method. The criteria for considering research data as valid include the completion of CBCT scans during each treatment fraction, with the confirmation of positioning error data by two experienced senior technologists. Notably, setup error data from patients in group A were excluded if the disparity between the measured bladder volume using BladderScan before treatment and the planned bladder volume exceeded 15%. In group A, there were 103 patient setup errors involving 15 patients, and a total of 114 patient setup errors were collected in group B. The patient setup errors were the displacement and rotation in all these directions including lateral (Lat), longitudinal (Lng), vertical (Vrt), Pitch, Yaw, and Roll.

The study was approved by the ethics committee of the First Affiliated Hospital of Soochow University. Written informed consent was obtained from each patient.

### 2.2. Planning CT and Setup Protocol

All patients were immobilized using vacuum bags. CT simulation scans were conducted using the Brilliance CT Big Bore system (Philips, Netherlands), with a slice thickness of 0.3 cm. To achieve a reproducible full bladder, patients of group A followed the protocol: The patients underwent urine and bowel emptying prior to the CT simulation scan and were then asked to drink 500–800 ml water. When the patient had the desire to urate, the current bladder capacity of the patient was measured with the ultrasonic BladderScan device BVI 9400 (Verathon Medical, Bothell, WA, USA), and planning CT was performed when the measured bladder urine volume reached more than 200–400 ml. The quantity of water consumed by the patient and the BladderScan reading were both documented. Patients in group B adhered to the instruction of consuming 400–500 mL of water and waiting for approximately 30 min before undergoing the CT simulation scan. Based on the setup protocol described above and considering historical departmental data on pelvic CBCT image matching errors, to prevent underdosage of CTV, the expansion margins for CTV-PTV are set as follows: 0.8 cm in the head-to-foot direction, 0.6 cm in the Vrt direction, and 0.6 cm in the Lat direction.

### 2.3. Patient Setup and Treatment

An Edge medical linear accelerator (Varian, Edge, CA, USA) is used at our center. The linear accelerator is outfitted with the OSMS (VisionRT, London, UK) system, facilitating real-time 3D surface imaging for precise patient positioning. The OSMS system is capable of capturing patient setup errors in Lat, Lng, and Vrt directions, as well as Pitch, Yaw, and Roll. The KV-CBCT system integrated within the linear accelerator enables image-guided radiotherapy (IGRT) for rectifying patient setup errors, encompassing corrections in Lat, Lng, and Vrt directions. Additionally, the PerfectPitchTM 6 degrees of freedom (6DoF) couch offers seamless adjustments across six axes, encompassing Pitch, Yaw, and Roll errors.

**Figure 1:**
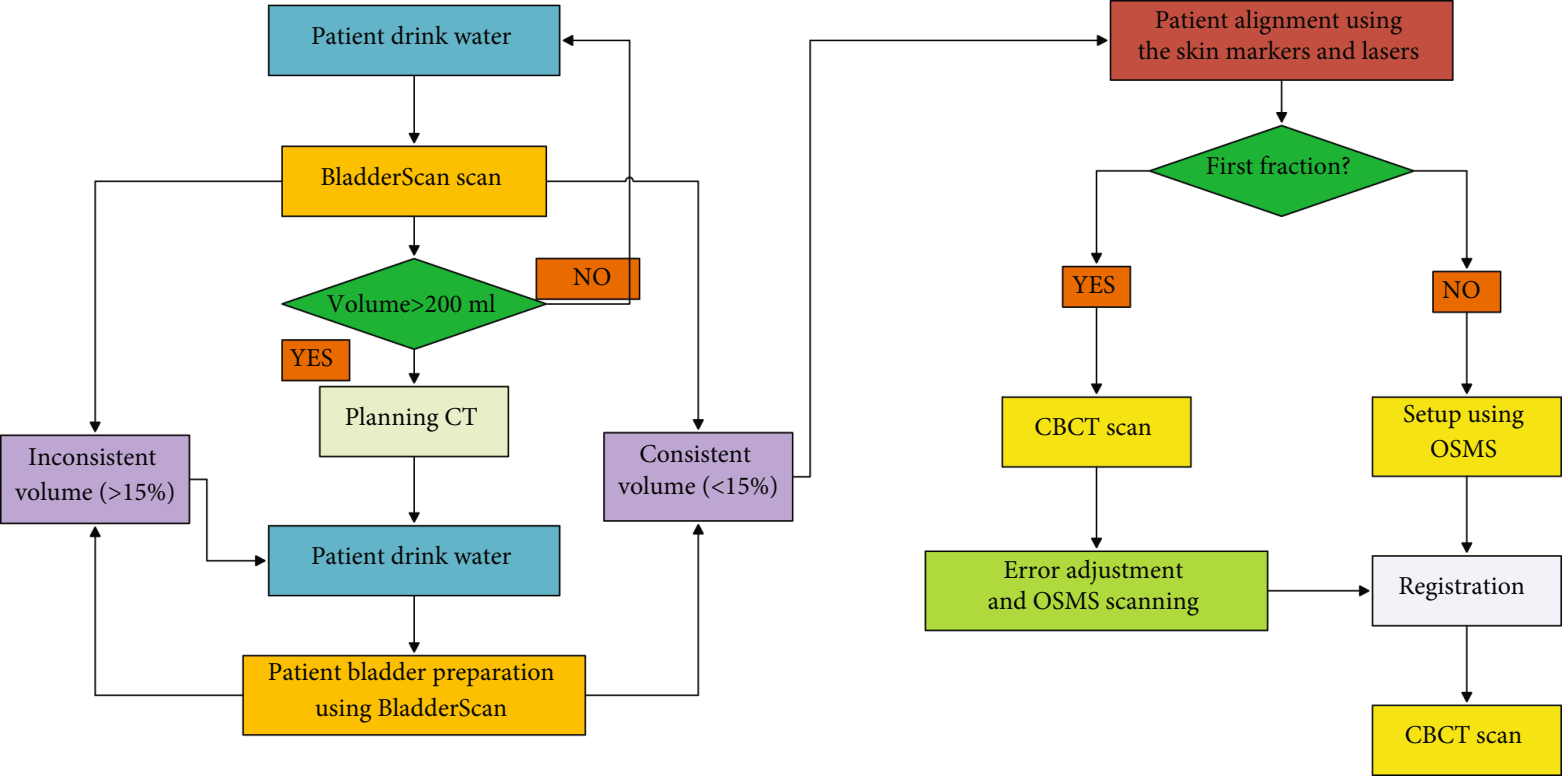
The planning CT and patient setup protocol of patients prior to treatment in group A.

Figure 1 illustrates the patient setup protocol for group A patients. Patients first underwent emptying of the bladder and bowels prior to treatment. Subsequently, patients were instructed to consume the same quantity of water as during the planning CT session. The current bladder capacity was then measured using BladderScan device BVI 9400. The BVI 9400 device offers three measurement modes: female, male, and children. For transabdominal scanning in the male mode, the scanning probe was positioned approximately 2 cm above the pubic symphysis, oriented at an angle towards the bladder. To ensure measurement accuracy, all measurements are conducted jointly by an experienced radiation oncologist and a radiographer. Upon establishing the accurate bladder position, bladder capacity was measured three times in succession. The resulting measurements were averaged to determine the bladder capacity. The deviation in bladder capacity between the measured and planned values was maintained within a controlled range of ±15%.

The years of experience of the two radiographers are as follows:

1. Radiographer: Hao Chen (12 years)
2. Radiation oncologists: Yandong Liu (7 years)

**Figure 2:**
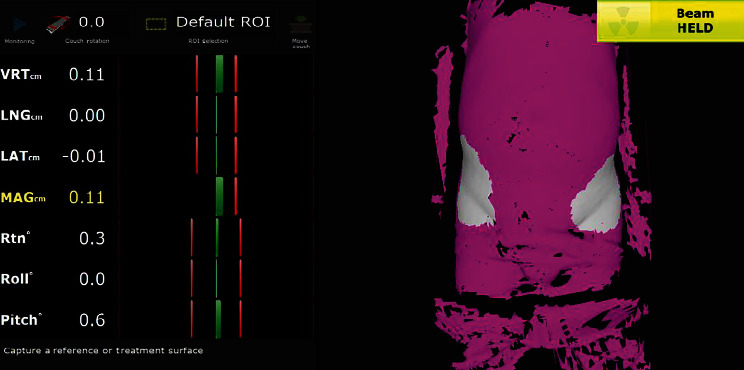
The white registration region of interest for OSMSref and the permissible limits for errors in Lat, Lng, Vrt, Pitch, Yaw, and Roll.

Prior to the initial treatment session, patients underwent a preliminary setup using skin markers. Subsequently, position verification was performed weekly using online KV-CBCT imaging. Initially, the CBCT images were aligned with the planning CT using an automatic bone-matching method. Following this step, a skilled senior technician carried out manual adjustments. Lastly, another senior technician cross-verified and validated the registration results. The permissible error limits for the Lng, Vrt, and Lat dimensions following patient CBCT registration are 0.8, 0.6, and 0.6 cm, respectively. Should these limits be exceeded, repositioning and a new CBCT scan are necessitated. The position was fixed with the error data generated by the system, the images of the patient surface were collected by the OSMS as reference images (OSMSref), and the region of interest (ROI) for OSMSref was selected as shown in Figure 2; for the white region, the ROI encompasses a region centered around the groin, extending 10–18 cm laterally and 5–15 cm longitudinally. During subsequent treatment sessions, optical surface images were captured to determine displacement in six axes: Lat, Lng, Vrt, Pitch, Yaw, and Roll. This was achieved through real-time registration with the ROI of OSMSref images. The permissible limits for errors in Lat, Lng, and Vrt were set at values lower than 0.3 cm. Additionally, the displacement of Pitch, Yaw, and Roll was restricted to less than 1 degree through patient repositioning. Verification of the setup was performed through scanning KV-CBCT images. Displacement measurements for Lat, Lng, and Vrt, as well as the rotation errors of Pitch, Yaw, and Roll, were documented following registration of the KV-CBCT images with the planning CT.

The patients in group B underwent spontaneous urine withholding, and the patient setup utilized the skin markers method. The displacements in Lat, Lng, and Vrt, along with the rotation errors of Pitch, Yaw, and Roll, were verified using KV-CBCT imaging.

### 2.4. Data Collection

A cumulative count of 235 CBCT images was amassed from both group A and group B. Prior to the commencement of the treatment, the technician conducted CBCT image scans to capture the patient's anatomical position during the current treatment fraction. Following registration with the planning CT, both patient positioning and rotation errors could be ascertained. The setup errors encompassing Lat, Lng, and Vrt, along with the rotation errors of Pitch, Yaw, and Roll, were recorded through CBCT registration in alignment with the planning CT. The CBCT images were transferred to the treatment planning system Eclipse13.6 (Varian, Eclipse, CA, USA), and two senior radiologists were invited to delineate the bladder on the CBCT images. The assessment of bladder consistency employed the Dice similarity coefficient (DSC) and quantified volume differences between CBCT and planning CT as evaluation parameters.

### 2.5. Data Analysis

All data were presented in terms of means and variances. The setup errors, DSC coefficients, and volume differences between groups A and B underwent statistical analysis through an independent sample *T* test. The statistical analysis was executed using SPSS 16.0 (IBM, Armonk, NY, USA). Two-sided *P* values were considered to determine statistical significance.

## 3. Results

The statistical results of setup errors are shown in Tables 1 and 2. The error data for Vrt, Lat, Lng, Pitch, Roll, and Yaw from both groups A and B were treated as absolute values, irrespective of the error's direction. As evidenced by Tables 1 and 2, group A exhibited lower setup errors compared to group B. The errors in group A at Vrt, Lat, and Lng were 0.17 ± 0.12, 0.22 ± 0.17, and 0.18 ± 0.12 cm, respectively, and in group B, they were 0.25 ± 0.15, 0.31 ± 0.21, and 0.34 ± 0.22 cm; the rotation errors of Pitch, Roll and Yaw in group A were 0.18 ± 0.12°, 0.11 ± 0.1°, and 0.18 ± 0.13° and in group B, they were 0.96 ± 0.89°, 1.01 ± 0.86°, 1.02 ± 0.84°. The statistical significance is denoted by *P* < 0.01 for the aforementioned performance indicators. Consequently, the patient setup approach integrating OSMS and BladderScan distinctly enhances accuracy.

**Figure 3:**
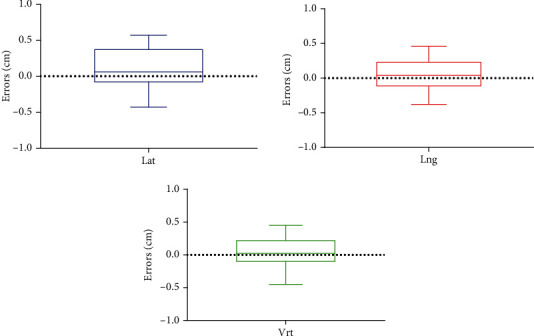
The boxplot of patient setup errors in the Vrt, Lat, and Lng directions for Group A.

Figure 3 displays the boxplot representing patient setup errors in the Vrt, Lat, and Lng directions for group A. The maximum patient setup errors between the anterior and posterior in the Vrt direction did not exceed 0.5 cm, and the interquartile range (IQR) spanned from −0.2 to 0.5 cm. The maximum patient setup errors did not exceed 0.5 cm in the Lat direction and the Lng direction, and the IQR of Lat and Lng all ranged from −0.3 to 0.5 cm. Nevertheless, the margin for CTV-PTV expansion ranges from 0.6 to 0.8 cm when employing the conventional patient setup method that depends on skin markers and spontaneous urine withholding. For all the cases selected in this study, the CTV-PTV margin expansion is 0.6 cm in the Vrt directions, 0.8 cm in the Lng directions, and 0.6 cm in the Lat directions. The results suggest that in group A, where patients employed the setup method combining BladderScan with OSMS, the CTV-PTV margin expansion in the Vrt, Lng, and Lat directions could potentially be reduced to 0.2–0.5 cm. This finding offers valuable guidance for clinical radiotherapy in postoperative prostate cancer, aiding in the refinement of CTV-PTV margin expansion.

Table 3 displays the statistical results comparing bladder volume differences and DSC coefficients between the planning CT and CBCT acquired prior to treatment for both group A and group B. The DSC of the bladder was 92.52 ± 1.65% in group A and 62.98 ± 22.33% in group B, and the volume difference was 39.99 ± 28.75 cm^3^ and 273.89 ± 190.62 cm^3^. The *P* < 0.01 of the above performance indicators indicates that the difference is statistically significant. Hence, patients in group A exhibited superior bladder consistency utilizing the protocol. Conversely, the protocol involving spontaneous urine withholding presented challenges in achieving consistent bladder filling reproducibility.

**Figure 4:**
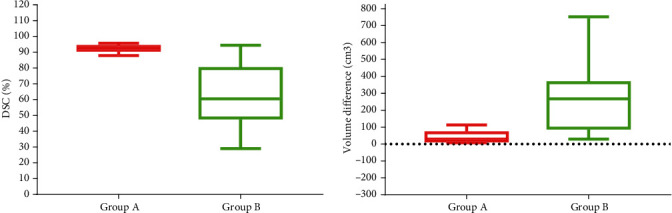
The DSC and volume difference results between CBCT and planning CT in group A and group B.

Further analysis reveals the depiction of the two indicators, DSC and volume differences for the bladder between CBCT and planning CT, in Figure 4. The findings suggest that in group A, the DSC ranged from 90% to 97%, while in group B, it varied from 43% to 95%. The IQR spanned from 91.38% to 93.8% in group A, and in group B, it was 48.49% to 79.78%. The boxplot illustrating bladder volume differences indicates a maximum difference of 110 cm^3^ in group A, with an upper quartile (Q3) of 66 cm^3^. In group B, the maximum difference was 750 cm^3^, and the Q3 was 362 cm^3^. Clearly, throughout the treatment fractionation, group A exhibited enhanced bladder volume consistency and smaller volume differences in comparison to group B.

**Figure 5:**
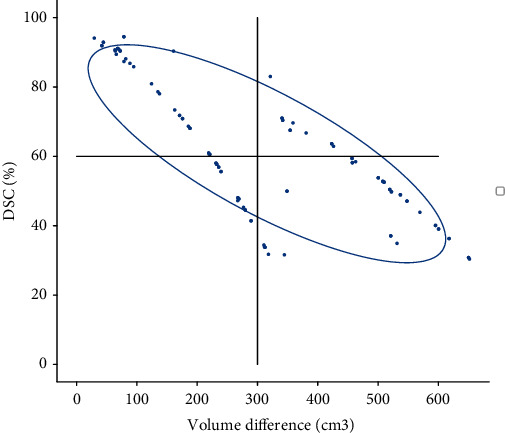
Distribution of the DSC and volume differences. The blue surface line shows the isoprobability surfaces that encapsulate 90% of the estimated multinormal probability distribution; a strong tendency of correlation between DSC and volume differences is seen.

Figure 5 is the ellipsoid that encapsulates 90% of the estimated pdf for DSC and volume differences, and the result showed that a strong correlation was found between the two indicators. On the *x*-axis, the bladder volume differences between CBCT and planningCT for the treatment fraction are depicted, while the *y*-axis represents the DSC of the bladder between CBCT and planningCT. Notably, a negative correlation exists: The smaller the bladder volume difference between CBCT and planning CT, the higher the DSC coefficient. This result furnishes substantial evidence emphasizing the imperative to minimize the disparity between planned and actual bladder volumes, ensuring accurate reproduction of the bladder in patients undergoing postoperative radiotherapy for prostate cancer.

## 4. Discussion

External beam radiotherapy for postoperative prostate cancer necessitates both consistent bladder filling and accurate patient positioning. The OSMS demonstrates promising accuracy and is radiation-free for patient setup. The BladderScan, utilized for bladder filling, offers specific advantages in maintaining a consistent and appropriately sized bladder volume [15]. However, there is a scarcity of studies on the combined application of OSMS and BladderScan in patient setup. Accordingly, this study sought to analyze 103 patient setup errors utilizing the combination of OSMS and BladderScan in a cohort of 15 postoperative prostate cancer patients. The findings demonstrated that the employed setup method serves as an effective tool to enhance both patient positioning accuracy and bladder reproducibility. The results indicate that the patient setup method, combining OSMS and BladderScan, can effectively diminish the CTV-PTV margin expansion in postoperative prostate cancer radiotherapy patients.

OSMS and BladderScan are two distinct tools that contribute to the precision of radiotherapy. While OSMS is widely utilized for the unmarked positioning and gating treatment of thoracic tumor patients, there exists relatively limited research on the application of OSMS in cases of abdominal tumors. Furthermore, in Pallotta's study, the advantages of OSMS in minimizing positioning errors were more pronounced for thoracic tumor patients (50%) than for abdominal tumor patients (45%) [16]. The primary factor influencing the positioning accuracy of OSMS in abdominal tumor cases is the lack of consideration for variations in bladder and rectal filling, which can impact the target area for these patients. BladderScan is frequently employed for abdominal tumor patients to assess bladder volume, replicate bladder volume during planning CT, and mitigate the risk of target underdosing and normal tissue overdosing attributed to irregular bladder filling. Building upon prior research on OSMS and BladderScan, this study expands the application of both tools in a synergistic manner for postoperative prostate cancer radiotherapy. Validating patient positioning errors against CBCT as the gold standard, the study contrasts the differences between the positioning methods presented in this paper and the conventional use of skin markers. The results indicate that the proposed method significantly reduces positioning errors within the range of 0.3–0.5 cm. Moreover, there is an improvement in the patient's rotation error. The study provided valuable insights for expanding CTV-PTV margin in postoperative prostate cancer radiotherapy.

The practice of patients voluntarily restraining urine is a widely employed strategy to maintain consistent bladder volumes during postoperative prostate cancer radiotherapy [17]. Nevertheless, reliance on this approach is susceptible to individual patient subjectivity, introducing disparities between the realized bladder volume and the intended volume. In group B, patients exhibited a maximum bladder volume discrepancy. Noteworthy is the substantial deviation of bladder volumes from the expected values during fractionated radiotherapy, resulting in a diminished DSC within this group. Within group A, a discernible enhancement in bladder volume consistency was observed during fractionated treatment in contrast to the initially planned bladder volume. Notably, the distribution of the DSC and volume differences distinctly illustrates that an escalation in bladder volume differences correlates with a reduction in the DSC coefficient for the bladder, denoting diminished bladder consistency. Our observations revealed that the mean Vrt errors ranged from 0.1 to 0.5 cm in group B. Partially, these errors can be attributed to patient abdominal bulging resulting from inconsistent bladder filling compared to the planning CT. Based on our experience, our study could represent a system to reduce setup errors and bladder changes without exposure to ionizing radiations and with a potential reduction of the cost of IGRT compared to the use of daily CBCT.

The PTV margin can be generally separated into two components, treatment preparation variations (delineation error and setup error) and day-to-day treatment variations (setup error and target motion) [18]. Two studies have addressed whether the application of BladderScan improved the procedure of bladder filling [19, 20]. Kuo et al. studied the effectiveness of BladderScan in reproducing bladder volume by comparing the measurements from BladderScan with the calculated volume from megavoltage computed tomography (MVCT), and the analysis of 314 sets of data from 11 patients indicates that the correlation coefficient between *V*_BS_ and *V*_CT_ was 0.87 [12]. Cramp et al. found that the BladderScan method for bladder filling was useful in achieving a consistent, appropriately sized bladder volume by accessing the passing rate of bladder requirements [20]. The study clearly demonstrates that, in contrast to group B where patients self-induced bladder filling, patients in group A, employing BladderScan for bladder volume control, manifested reduced disparities between the actual bladder volume and the volume outlined in the planning CT. During fractionated treatments, group A exhibited a higher DSC for the bladder, signifying a significant resemblance to the bladder outlined in the planning CT. The obtained results are in line with prior research. Additionally, this study identified a robust correlation between bladder volume differences and the DSC for the bladder. These novel findings indicate that BladderScan serves as an effective measurement tool for replicating bladder volume, improving the DSC coefficient between the bladder and planned CT during fractionated treatments, thus fulfilling the prerequisites for accurate radiotherapy.

OSMS represents an innovative markerless radiotherapy patient setup method. In contrast to the conventional skin marker, OSMS provides enhanced positioning accuracy. This method is progressively gaining traction in patient radiotherapy setup owing to its inherent advantages [21]. A study by Ma reported the OSMS for patient positioning in interfractional breast cancer radiotherapy, and the results showed good agreement between OSMS and CBCT. For the 200 setups, the interfractional displacements on the Lat, Lng, and Vrt directions for OSMS were 0.049 ± 0.254, 0.018 ± 0.261, and 0.062 ± 0.254 cm, respectively, and the maximum interfractional displacement in the Vrt, Lng, and Lat directions are within 0.5 cm [22]. Moreover, Pallotta et al. conducted a comparative analysis of surface imaging, portal imaging, and skin marker setup in thoracic and pelvic radiotherapy, employing CBCT data as the reference standard [16]. The study encompassed 20 patients, evaluating the efficacy of surface imaging in comparison to a skin marker setup. The optimized patient positioning was advantageous in 45% of pelvic fractions. The displacements in the Lat, Lng, and Vrt directions were measured at 0.01 ± 0.25, −0.14 ± 0.4, −0.16 ± 0.31, and 0.07 ± 0.21 cm, respectively. Consistently, the current study found that errors in CBCT scanning within group A in the 3D direction were improved and consistently below 0.5 cm, aligning with findings from prior studies. This observation indicates that OSMS can serve as an effective strategy to enhance the precision of patient positioning in postoperative prostate cancer radiotherapy.

Our study acknowledges several limitations: (1) the concurrent use of OSMS and BladderScan leads to extended patient setup times. The presence of hair in the pelvic perineum area can impact the accuracy of OSMS; the existence of hair diminishes the information available for optical systems, and depilation may be beneficial in addressing this issue. The accuracy of optical systems is influenced by the intensity of light; accumulation of fat in the abdomen may occur, and the ROI should be carefully defined to exclude tissues prone to deformation or movement, such as muscles, skin folds, and respiratory motion, in order to minimize positioning errors. OSMS, as an emerging technology, necessitates meticulous equipment quality assurance, comprehensive end-to-end process testing, and a thorough risk assessment of failure models before implementation in clinical and scientific settings. Consequently, departments should establish a systematic and standardized set of procedures. (2) Patients, irrespective of age, exhibit varying urinary retention capabilities, and urinary secretion is influenced by factors such as diet, fluid intake, and patient age. The onset of a “sense of urgency” varies among individuals, necessitating multiple measurements in clinical practice. The BladderScan BVI9400, introduced in 2007, provides positioning accuracy of approximately 15%, with diverse selection conditions. Proficiency in operator technique is crucial due to its varied operational requirements. Crop et al. reported overall low consistency between measurements from the bladder scanner and CT scans in the upright position, with 95% limits of agreement (LOAs) exceeding ±200 cm3 [23]. This inconsistency is attributed to poor patient repeatability and the presence of fixed and proportional biases. Correction for these biases can only be achieved under optimal conditions, involving male patients in a supine position with three bladder scan measurements taken. (3) In addition, our study retrospectively analyzed the overall setup errors of the 2 methods and did not analyze the interfractional setup errors of patients. A study by Apicella et al. investigated intrafraction setup variations in the pelvic district acquiring multiple body surface images captured by a noninvasive 3D surface imaging system during each radiotherapy session [24]. The result showed that the dominant direction in setup variations was vertical with mean values of −0.12 ± 0.006 cm and the longitudinal mean components were −0.095 ± 0.010 cm while the lateral deviations were 0.007 ± 0.008 cm. Therefore, in the follow-up work, it is necessary to study the interfraction error of this setup method. The sample size was small, and all patients were from the same hospital, thus possibly introducing some bias and the need to collect more sample data to guide CTV-PTV margins.

In recent years, several studies have utilized OSMS for patient verification [16, 25]. Although these systems have found broader applications in breast, chest, and limb tumors, their use in pelvic tumors has been relatively limited. Notably, there is a paucity of research combining OSMS with BladderScan. The patient setup method, integrating OSMS and BladderScan, confers several advantages in this investigation: (1) Adjustability with OSMS: OSMS allows for adjustments in patient setup both before and after immobilization, facilitating the correction of translational and rotational errors. It dynamically monitors changes in position throughout the treatment process. In contrast to skin markings, OSMS offers real-time monitoring of thousands of geometric nodes on the body surface, providing more detailed information. This capability enables precise detection of pelvic rotation and hip joint positions, particularly important given the susceptibility of the hip joint to rotation. (2) Real-time monitoring with OSMS: Before treatment initiation, OSMS can visually capture the six-axis positioning errors of the patient. By adjusting the patient's position based on this information, the positioning errors can be reduced to within the required range. (3) Efficiency: Research indicates that the scanning duration for CBCT is approximately 60 s [22]. The processes of image processing, matching, and couch displacement collectively demand a minimum of 5 min. Conversely, OSMS has an average scanning time of under 20 s, contributing to a total positioning time of less than 1 min [26]. (4) Bladder consistency with BladderScan: The implementation of the BladderScan feedback method facilitates patients in achieving optimal bladder filling, thereby enhancing the success rate in reproducing the bladder volume as delineated in the planning CT. (5) Zero radiation: In our investigation, the OSMS employs visible light projection onto the body surface, thereby avoiding any contribution to the patient's radiation dose. The BladderScan utilizes ultrasound for bladder volume measurement, eliminating patient exposure to radiation. This radiation-free attribute is crucial in safeguarding patients from additional radiation harm throughout the treatment process. Radiographic imaging methods used for position verification include CBCT, 2D X-ray imaging, and low-dose CT scans. However, these imaging modalities expose patients to additional radiation doses during their treatment. The radiation dose within the imaged area during CBCT usage for radiation therapy has been extensively documented, and studies have reported measurements of patient dose. The typical dose of the isocenter for a single CBCT is 0.015–0.03 Gy [27–29]. The risk of cancer induced by ionizing radiation has been confirmed, and the risk of a secondary cancer is greatly increased by radiotherapy [30, 31].

## 5. Conclusions

Inconsistent dose distribution during postoperative prostate cancer radiotherapy is predominantly attributed to patient setup errors and bladder variations. The approach of combining OSMS and BladderScan exhibited favorable outcomes in minimizing setup errors within our study. The daily variations detected using our method within our series align with existing literature data, while also revealing noteworthy distinctions in minimizing Lat, Vrt, Roll, and Yaw errors when compared to studies relying solely on SGRT positioning. The primary explanation for this divergence can be attributed to the synergistic utilization of BladderScan and presetup with OSMS. Consequently, these findings propose that this technique could serve as a daily, cost-effective, and nonradioactive setup modality for postoperative prostate cancer patients.

## Figures and Tables

**Table 1:** The statistical results of Vrt, Lat, and Lng between group A and group B.

**Parameter**	**Vrt (cm)**		**Lat (cm)**		**Lng (cm)**	
	**Group A**	**Group B**	**Group A**	**Group B**	**Group A**	**Group B**
Mean	0.17	0.25	0.22	0.31	0.18	0.34
Standard deviation	0.12	0.15	0.17	0.21	0.12	0.22
*P* value	*P* ≤ 0.001		0.02		*P* ≤ 0.001	

**Table 2:** The statistical results of Pitch, Roll, and Yaw between Group A and Group B.

**Parameter**	**Pitch (°)**		**Roll (°)**		**Yaw (°)**	
	**Group A**	**Group B**	**Group A**	**Group B**	**Group A**	**Group B**
Mean	0.18	0.96	0.11	1.01	0.18	1.02
Standard deviation	0.12	0.89	0.1	0.86	0.13	0.84
*P* value	*P* ≤ 0.001		*P* ≤ 0.001		*P* ≤ 0.001	

**Table 3:** The statistical results of DSC and volume difference of bladder between the planning CT and CBCT for group A and group B.

**Parameter**	**DSC (%)**		**Volume difference (cm** ^**3**^ **)**	
	**Group A**	**Group B**	**Group A**	**Group B**
Mean	92.52	62.98	39.99	273.89
Standard deviation	1.65	22.33	28.75	190.62
*P* value	*P* ≤ 0.001		*P* ≤ 0.001	

## Data Availability

The datasets used and/or analyzed during the current study are available from the corresponding author on reasonable request.
